# Geographic Variation in Venom Proteome and Toxicity Profiles of Chinese *Naja atra*: Implications for Antivenom Optimization

**DOI:** 10.3390/toxins17080404

**Published:** 2025-08-12

**Authors:** Jianqi Zhao, Xiao Shi, Guangyao Liu, Yang Yang, Chunhong Huang

**Affiliations:** 1 Physical Examination Center, The Second Affiliated Hospital of Nanchang University, Nanchang University, Nanchang 330031, China; zhaojq2001@163.com; 2 School of Basic Medical Sciences, Jiangxi Medical College, Nanchang University, Nanchang 330031, China; 3 Jiangxi Provincial Key Laboratory of Tumor Biology, Nanchang University, Nanchang 330031, China; jp4217122156@qmul.ac.uk (X.S.); joshualiu2002@163.com (G.L.); 4 Queen Mary School, Jiangxi Medical College, Nanchang University, Nanchang 330031, China; 5 The First Clinical Medical College, Jiangxi Medical College, Nanchang University, Nanchang 330031, China

**Keywords:** snake venom, *Naja atra*, PLA2, 3FTX, antivenom, proteomics

## Abstract

Differences in venom within snake species can affect the efficacy of antivenom, but how this variation manifests across broad geographical scales remains poorly understood. *Naja atra* envenoming causes severe morbidity in China, yet whether intraspecific venom variation exists across mainland regions is unknown. We collected venom samples from seven biogeographical regions (spanning > 2000 km latitude). Venom lethality, systemic toxicity (organ damage biomarkers and coagulopathy), and histopathology of major organs were assessed. Neutralization by antivenom and label-free quantitative proteomics (LC-MS/MS) were also performed. The results revealed a non-uniform LD_50_, with venom from Yunnan exhibiting the highest lethality (2.1-fold higher than venom from Zhejiang, *p* < 0.001). Commercial antivenom showed lower neutralization efficacy against the venom from the Yunnan, Guangxi, and Guangdong regions. Regarding organ damage and coagulopathy, venom from Yunnan caused severe liver damage, while venom from the Zhejiang region induced significant coagulopathy. Finally, proteomic profiles identified 175 proteins: venom from Yunnan was dominated by phospholipases, contrasting with eastern regions (Anhui/Zhejiang: cytotoxins CTXs > 30%). Venom from Guangdong contained higher levels of the weak neurotoxin NNAM2 (5.2%). Collectively, significant geographical divergence exists in Chinese Cobra venom composition, systemic toxicity, and antivenom susceptibility, driven by differential expression of key toxins. Our study provides a molecular basis for precision management of snakebites, and we call for optimized antivenom production tailored to regional variations.

## 1. Introduction

Venom is an evolving weapon system in snakes, exhibiting significant intraspecific variation across geographical ranges [1]. This plasticity poses major challenges for the clinical management of envenomation, as commercial antivenoms prepared against venoms from specific locales often exhibit variable efficacy against different populations [2,3,4,5]. Venom diversity and variation are increasingly documented, but research in Asia lags [6,7,8,9]. Medically, understanding venom variation is crucial for developing region-specific antivenoms and establishing clinical management strategies for snakebites.

*Naja atra* is one of China’s most medically significant venomous snakes, widely distributed across southern and southeastern China. Its venom is a complex mixture of bioactive components, primarily comprising neurotoxins, cytotoxins, and diverse enzymes [10,11,12]. These venom constituents act synergistically to induce multiple toxic effects in envenomed victims, including neurotoxicity, local tissue damage, coagulopathy, and cardiotoxicity [13,14,15]. The high morbidity and mortality resulting from Chinese *Naja atra* bites underscore the importance of understanding its venom properties and developing effective countermeasures. Although intraspecific venom variation has been confirmed in some snakes globally [2,16], analyses of such variation in Chinese *Naja atra* venom remain extremely limited.

While proteomic studies have identified phospholipase A_2_ (PLA_2_), three finger toxins (3FTx), and snake venom metalloproteinases (SVMP) as key toxic components responsible for the pathogenicity of *Naja atra* venom [17,18,19], it is noteworthy that subtype diversification within toxin families may determine venom lethality and organ-specific injury patterns. However, whether such compositional heterogeneity exists across China’s complex biogeographical regions—from subtropical forests to high-altitude plateaus—remains unknown. Previous studies were limited in scope. To address this knowledge gap regarding the geographic variability of *Naja atra* venom in China, we conducted a systematic investigation of venoms from seven representative Chinese regions (Yunnan (YN), Anhui (AH), Zhejiang (ZJ), Jiangxi (JX), Guangxi (GX), Guangdong (GD), and Hunan (HN)). These provinces span nearly 2000 km in latitude. We analyzed venom lethality, systemic toxicity, multiorgan damage potential, and proteomic profiles. We demonstrate: 1. Significant regional variation in venom potency, with YN being the most potent and ZJ the least; 2. The relationship between differential antivenom neutralization effects and proteomic signatures; 3. Variability in organ-specific toxicity patterns and coagulation parameters linked to venom geographic origin; 4. Distinct geographic venom proteomic fingerprints.

Our findings provide novel insights into the geographical divergence of *Naja atra* venom. Understanding regional variation in cobra venom contributes to insights into the species’ evolutionary biology and ecological factors influencing venom evolution. Medically, comprehending venom variation is essential for developing region-specific antivenom therapies. Currently, the therapeutic efficacy of antivenom against *Naja atra* envenoming may be limited because of the compositional differences in venoms from distinct geographic regions. A deep understanding of this variation may facilitate the development of more targeted and effective antivenoms, thereby improving clinical management outcomes for snakebite patients.

## 2. Results

### 2.1. Regional Differences in Venom LD_50_

We systematically determined the LD_50_ of *Naja atra* venom from seven representative geographical regions in China by intraperitoneal injection into mice. The results revealed significant geographical variation in the lethal toxicity of venoms from different regions (Table 1). Venom sourced from YN exhibited the strongest lethal toxicity, followed by GX and GD, while ZJ-sourced venom showed relatively the weakest toxicity. Notably, venoms from HN, JX, and AH demonstrated moderate lethal toxicity. Overall, the results showed marked differences in venom across regions.

### 2.2. Geographic Variation in Systemic Toxicity and Multiorgan Damage Induced by Venom

**Figure 1:**
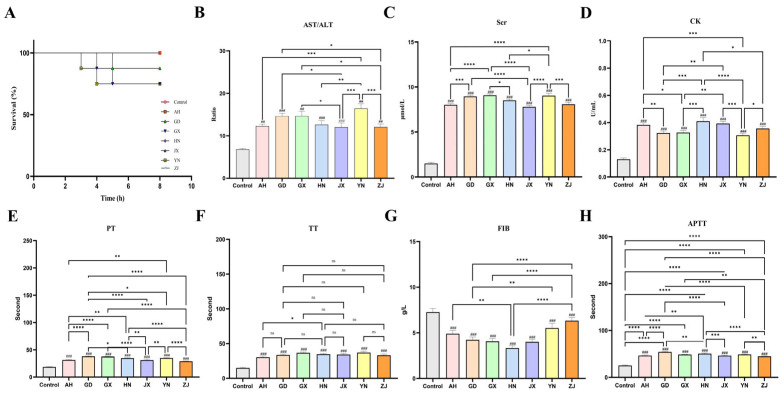
Antivenom neutralization efficacy and venom-induced systemic damage. (**A**) The neutralizing effect of equal amounts of antivenom serum on different venoms. (**B**–**D**) These are the indicators for detecting liver (AST/ALT), kidney (Scr), and heart (CK) damage. (**E**–**H**) Coagulation function test. Error bars represent the standard error of the mean for *n* = 3 replicates (^ns^ *p* > 0.05, * *p* ≤ 0.05, ** *p* ≤ 0.01, *** *p* ≤ 0.001, **** *p* ≤ 0.0001, ## *p* ≤ 0.01 and ### *p* ≤ 0.001).

Regarding antivenom neutralization (Figure 1A), the neutralization efficacy of equivalent antivenom doses differed across venoms. Neutralization capacity was weaker against YN, GD, and GX venoms, consistent with the LD_50_ results. This may be related to differences in venom enzymatic activity or composition. For organ damage indicators (Figure 1B–D), liver function, kidney function, and cardiac injury biomarkers demonstrated significant variations among venoms, with certain venoms causing more pronounced damage. Coagulation function tests further revealed geographical divergence: prothrombin time (PT), thrombin time (TT), fibrinogen concentration (FIB), and activated partial thromboplastin time (APTT) showed variations in some venoms, indicating differing capacities of venoms to disrupt coagulation (Figure 1E–H). Collectively, these results demonstrate that venoms from different Chinese regions exert significantly different systemic toxicities in mice.

**Figure 2:**
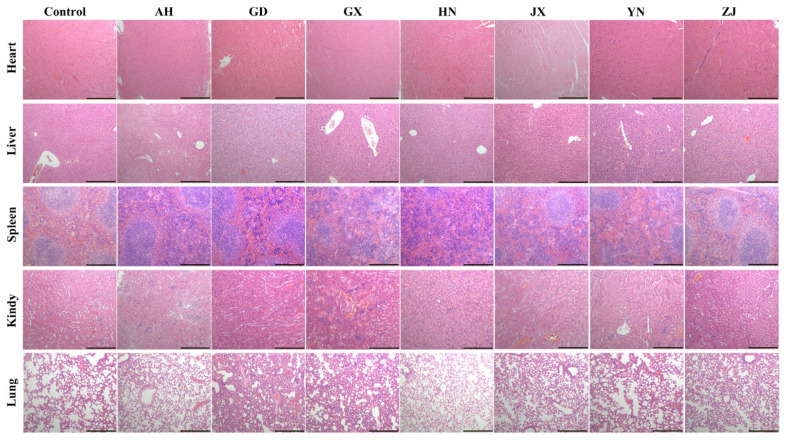
Histopathological variations induced by geographically distinct *Naja atra* venoms across China. After injecting mice with snake venom from different geographical sources, the hearts, livers, lungs, kidneys, and spleens were collected and stained with hematoxylin and eosin. The picture shows the most representative result. Scale bar: 500 μm.

Histopathological assessment revealed significant geography-dependent organ damage patterns, highly consistent with venom protein composition and serum biomarkers. Venoms from different regions induced characteristic organ lesions (Figure 2). YN venom caused the most extensive damage, manifesting as myocardial fiber rupture, hepatocyte ballooning degeneration, and acute renal tubular necrosis (brush border loss/cast formation). GX and GD venoms primarily induced splenic white pulp atrophy and focal pulmonary hemorrhage. AH and ZJ venoms featured prominent wavy myocardial fiber degeneration (consistent with CTX-3 cardiotoxicity), with milder liver/kidney injury (lesion area < 15%). JX and HN venoms presented moderate renal cortical perivascular edema. These results confirm that differences in venom composition directly determine pathological phenotype heterogeneity.

### 2.3. Proteomic Profiling Reveals Geographical Variation in Naja atra Venom Composition

**Figure 3:**
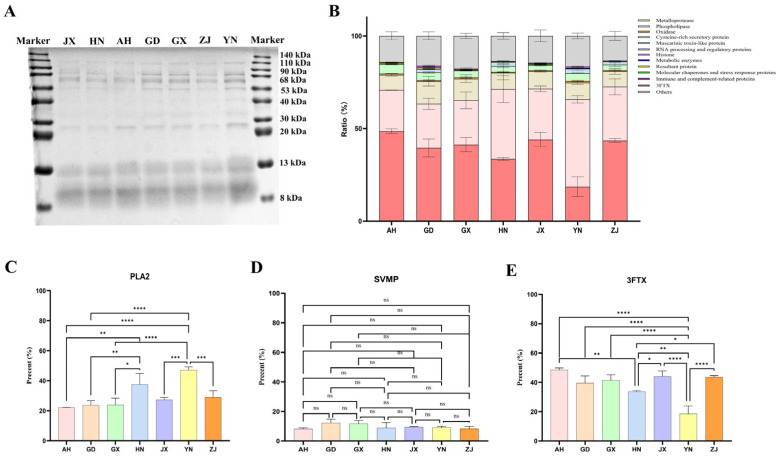
Proteomic analysis of *Naja atra* venom from different geographic regions. (**A**) Coomassie blue staining analysis of venom samples from seven different regions in China. (**B**) Bar graph representing the relative protein content in venom samples from different regions. The graph is divided into sections representing different protein families, with the color coding shown in the legend on the right. (**C**–**E**) Bar graph showing the relative content of PLA2, SVMP, and 3FTX in venom samples from different regions. Error bars represent the standard error of the mean for *n* = 3 replicates (^ns^ *p* > 0.05, * *p* ≤ 0.05, ** *p* ≤ 0.01, *** *p* ≤ 0.001, and **** *p* ≤ 0.0001).

Coomassie blue staining (Figure 3A) preliminarily revealed differences in the electrophoretic profiles of *Naja atra* venoms across regions. The marker indicated a molecular weight range from 140 kDa to 8 kDa, showing variations in band distribution and intensity across different molecular weight regions among regional venoms. For example, in the high molecular weight (>68 kDa) region, ZJ and YN samples exhibited relatively distinct and intense bands; in the medium-low molecular weight regions (30–68 kDa and <30 kDa), samples such as AH and GD displayed distinct band features, providing a macroscopic basis for subsequent protein component analysis. Using liquid chromatography–tandem mass spectrometry (LC-MS/MS) quantitative analysis, proteomic identification was performed on *Naja atra* venoms from these seven geographical regions in China (Anhui, Guangdong, Guangxi, Hunan, Jiangxi, Yunnan, Zhejiang). A total of 175 proteins were identified (Table 2). Further analysis resolved the geographical differences in the proportion of major protein families (Figure 3B–E) and key proteins (PLA_2_, SVMP, 3FTx). From the overall distribution of protein families, the proportions of various proteins (e.g., phospholipases and three finger toxins) differed in venoms from different regions.

As shown in Table 2, further analysis of core toxin families and their geographical distribution revealed that 3FTx exhibited the highest abundance and most significant diversity, with CTXs being the most prominent. CTX 3 dominated in multiple venoms, especially in AH (29.251%) and ZJ (30.163%), while being significantly lower in YN (6.192%). CTX 2 was most abundant in JX (5.856%); CTX 6 was prominent in AH (3.321%) but low in other regions; CTX 4N was present in GD (1.456%), HN (1.174%), and YN (1.025%) venoms. The weak neurotoxin NNAM2 was relatively higher in GD (5.167%) and HN (4.336%), though still at low levels. Weak toxins (e.g., weak toxin CM-11, weak neurotoxin 7, and weak neurotoxin 6) and neurotoxins (e.g., long neurotoxin 1 and α-synaptotoxin Nala) were ubiquitous but exhibited region-dependent abundance variations.

In the phospholipase family, the neutral phospholipase A_2_ muscarinic inhibitor (Q92084) was one of the highest-abundance single proteins, particularly prominent in YN (41.201%). Acidic phospholipase A_2_ (P00596) was widely highly expressed, with peak levels in HN (11.574%) and YN (5.862%). Basic phospholipase A_2_ (P00595) was low in abundance but detectable in YN (0.070%) and ZJ (0.082%); other isoforms (e.g., A0A898INR6) were generally below 0.05%. Snake venom metalloproteinases (SVMPs), while exhibiting interregional diversity in subtypes, showed no significant differences in total abundance across venoms.

Beyond core toxins, other bioactive components were identified. For example, Cysteine-rich secretory proteins showed abundance gradients significantly correlated with geography. L-amino acid oxidase (LAAO) was ubiquitous (0.481–0.901%) with minor regional variation. Venom nerve growth factor (Q5YF89) was stably expressed (0.423–0.767%). Snake venom 5′-nucleotidase (A0A194AS98) was significantly enriched in YN (1.779%). Collectively, the relative abundance of core toxins showed significant geographical divergence. Key isoforms were specifically enriched in regional venoms: e.g., neutral PLA_2_ (41.201%) in YN; CTX 3 (>29%) in AH and ZJ; weak neurotoxin NNAM2 (5.167%) in GD. Abundances of CRISPs, CVF, and C-type lectins also varied with geography. These compositional differences likely constitute the key molecular basis for the observed regional variations in LD_50_ and murine injury phenotypes.

## 3. Discussion

This study is the first to systematically reveal significant geographical variation patterns in lethal toxicity (LD_50_), induction of systemic multiorgan damage characteristics, and antivenom neutralization efficacy of Chinese *Naja atra* venom. This variation is not random but exhibits a clear gradient change from west to east (Yunnan → Guangxi/Guangdong → Hunan/Jiangxi/Anhui → Zhejiang), with its molecular basis rooted in the regional adaptation and differentiation of the venom proteome.

Yunnan (YN) venom exhibited the strongest lethal toxicity (LD_50_ 0.23 mg/kg), significantly higher than Zhejiang (ZJ) venom (difference exceeding 2.1-fold, *p* < 0.001). Proteomic analysis provided a key explanation for this phenomenon: it may be related to the abundance of PLA in YN venom. This toxin possesses potent myotoxicity and cytotoxicity, capable of inducing severe multiorgan failure (manifested as sharp increases in ALT/AST and Cr levels) in organs such as the liver and kidney through triggering mitochondrial apoptosis and cytomembrane lysis [12,20,21,22]. Conversely, venoms from eastern regions (e.g., AH and ZJ) are dominated by 3FTx, particularly CTX. Although these toxins cause relatively low systemic lethality, they can rapidly block neuromuscular junctions and myocardial potassium channels, leading to swift paralysis and significant myocardial damage [23,24,25,26]. JX venom exhibited a unique tendency for coagulopathy (significantly prolonged PT), which may be related to its specific spectrum of snake venom metalloproteinases (SVMPs) and C-type lectins [27,28]. This pattern of “western cytotoxicity (PLA_2_-dominant) versus eastern neurotoxicity (3FTx-dominant)” likely reflects adaptive evolution to local ecological environments and prey composition: the high-altitude regions of Yunnan are dominated by rodents requiring rapid tissue dissolution for subjugation, while eastern regions feature amphibian prey reliant on neuroparalytic for control [29,30,31]. Genetic evidence (e.g., approximately 4% divergence in the cytochrome c oxidase subunit I (COI) gene in Chinese cobra) further supports the hypothesis that populations may form adaptive evolutionary lineages [32].

A clinically significant finding is that commercially prepared antivenom exhibits significantly reduced neutralization efficacy against venoms from Yunnan, Guangxi, and Guangdong. According to proteomic results, this inefficacy may stem from molecular mismatches and antigen deficiency in venom components. Yunnan-specific CTX-4N and SVSP, as well as weakly neurotoxic NNAM2 enriched in Guangdong, are likely present in extremely low quantities or entirely absent in existing antivenom immunogens, resulting in insufficient neutralization capacity against these “endemic toxins.” It may also relate to the formation of heterocomplexes by PLA_2_-3FTx, which could alter individual toxin epitopes and reduce antibody recognition efficiency [33,34,35].

The findings urgently necessitate a shift from the current “one antivenom for multiple regions” strategy toward geographically precise viper bite treatment. Treatment strategies should be regionally tailored. For high PLA_2_/high systemic toxicity risk areas (e.g., Yunnan, Guangxi, and Guangdong), prioritize PLA_2_ inhibitors (e.g., varespladib) as adjuvant therapy alongside antivenom [36,37,38,39], and accelerate development of next-generation antivenoms effectively covering region-specific toxins (e.g., CTX-4N and NNAM2). For high cardiotoxicity risk areas dominated by CTX (e.g., Anhui, Zhejiang), rigorous cardiac function monitoring is critical. For regions exhibiting coagulopathy (e.g., Jiangxi), stockpile corresponding coagulation factor supplements. Developing multiplex immunoassays (e.g., targeting PLA_2_/CTX-3 ratios) for rapid geographical identification of envenoming sources may be pivotal for achieving precise triage and treatment [40].

Although this study outlines the primary framework of geographical variation in Chinese *Naja atra* venom, limitations remain. This study did not account for onto-genetic variation (juvenile vs. adult) in venom. While key geographical signature toxins (e.g., YN-PLA_2_ and AH-CTX3) were identified, their specific contributions and synergistic effects in particular pathological injuries (such as liver/kidney failure and coagulopathy) require further confirmation through functional studies. Geographical coverage could be expanded, including Fujian, Hainan, and other regions with unique ecological niches. Furthermore, certain human factors exist during venom collection. Additionally, due to government prohibitions on killing wild snakes, venom glands could not be obtained for RNA-seq, preventing matching with proteomic data. Despite these limitations, our conclusions remain unaffected. Future research should focus on integrating venom gland transcriptomics and genomics to comprehensively analyze the molecular mechanisms of toxin geographical differentiation, validate venom pathological phenotypes in humanized models to enhance the reliability of clinical translation, and collaborate with antivenom manufacturers to rigorously evaluate and optimize the neutralization breadth of region-specific or polyvalent antivenoms prepared based on geographical venom banks.

## 4. Conclusions

In summary, Chinese *Naja atra* venom is not homogeneous but constitutes a complex geographical toxicity mosaic. Behind the “cytolytic storm” of western (Yunnan) venom and the “neuroparalytic edge” of eastern (Zhejiang) venom lies a differential molecular strategy dominated by PLA_2_ and 3FTx. This is a result of ecological adaptation and profoundly influences clinical outcomes. The neutralization “blind spots” of antivenom reveal the severe reality of molecular mismatch. This study provides a systematic paradigm for understanding venom geographical variation in *Naja atra*, and more importantly, lays a solid scientific foundation for advancing geographically precise snakebite prevention and control in China based on venom origin.

## 5. Materials and Methods

### 5.1. Snake Venom

The venom was collected by professional personnel from the Jingdezhen Snake Farm (Jingdezhen City, Jiangxi Province, China) in China’s Yunnan, Anhui, Zhejiang, Jiangxi, Guangxi, Guangdong, and Hunan provinces. A total of 5 *Naja atra* specimens were collected from each region. Referencing the previous literature, the snake venom was encouraged to penetrate through a membrane covered with a cellulose film and enter a 50 mL centrifuge tube. Then, the venom obtained from individual snakes in the same region was combined, freeze-dried, and stored at −20 °C before use [41].

### 5.2. Animals Model and Ethics

Kunming mice (male, approximately 25–35 g, 6–8 weeks of age) were obtained from the Animal Center of Nanchang University (Nanchang, Jiangxi Province, China). According to procedures previously described [15], each experimental group comprised six mice, with a total of eight groups in this study. (a) Normal saline (Control group); (b) snake venom from Anhui (AH group); (c) snake venom from Guangdong (GD group); (d) snake venom from Guangxi (GX group); (e) snake venom from Hunan (HN group); (f) snake venom from Jiangxi (JX group); (g) snake venom from Yunnan (YN group); (h) snake venom from Zhejiang (ZJ group). Based on the results of the experiment in Section 5.3, the dosage was 0.2 times the LD_50_ of the venom. The venom was administered by intraperitoneal injection. Twenty-four hours later, under isoflurane anesthesia, blood samples were collected from the orbital area (500–600 μL for each mouse) by the ocular puncture method. Blood was transferred to heparin-coated tubes, centrifuged at 3000 rpm for 10 min at 4 °C, and serum aliquots were stored at −80 °C. Subsequently, the cervical vertebrae were dislocated, and the mice were euthanized, and the main organs were collected. The serum and organs of the mice were collected for subsequent experiments. All animal experiments were conducted in accordance with the guidelines for animal experiments at Nanchang University and protocols approved by the Nanchang University Animal Ethics Committee (Ethics code: NDSYDWLL-202131).

### 5.3. LD_50_ Test

The median lethal dose (LD_50_) was determined via intraperitoneal injection in specific pathogen-free male mice (*n* = 6 per dose group). Venoms from each region were dissolved in sterile saline (0.9% NaCl) and serially diluted across seven concentrations (0.1–0.7 mg/kg body weight) determined by preliminary range-finding tests. Each mouse received a fixed injection volume of 0.1 mL. The control group received an equivalent volume of saline. Mortality was recorded at 24 h post-injection. Repeat the above method three times, and the LD_50_ was calculated using the Spearman–Karber method [41].

### 5.4. Determination of Neutralizing Efficacy of Antivenom Serum

The neutralization efficacy of antivenom was evaluated using an in vitro pre-incubation model [36]. Each venom was dissolved in sterile physiological saline. Based on preliminary experiments, 100 µL of venom solution (venom concentration: 1.26 mg/kg) was injected per mouse. Twice the volume of commercially available equine polyvalent Zhejiang cobra antivenom (Specification: 10,000 U/mL; Lot No.: 20230307; Expiry Date: 2026.06; Shanghai Serum Bio-technology Co., Ltd., Shanghai, China) was added to the venom solution, and the mixture was pre-incubated at 37 °C for 10 min. The mixture was then administered to mice (*n* = 8/group) via intraperitoneal injection. Mortality was recorded over a continuous 24 h observation period.

### 5.5. Ser-Enzyme Assays

Commercial kits (Nanjing Jiancheng Biological Engineering Institute, Nanjing, China) were used to analyze previously collected serum. The parameters analyzed included alanine aminotransferase (ALT), aspartate aminotransferase (AST), creatine kinase (CK), and serum creatinine (Scr).

### 5.6. Evaluation of Hemostatic Parameters

The measurements of fibrinogen concentration (FIB), prothrombin time (PT), thrombin time (TT), and activated partial thromboplastin time (APTT) were determined using commercial kits (Rayto Life and Analytical Sciences^®^, Shenzhen, China) and an automatic coagulation analyzer (Servicebio^®^, Wuhan, China) according to the manufacturer’s protocols.

### 5.7. Histological Analysis

Histopathological analysis was conducted based on previous studies with modifications [15]. Tissue samples were fixed in 4% paraformaldehyde buffer for at least 24 h, dehydrated in ethanol, embedded in paraffin, and cut into 5 µm sections. Sections were deparaffinized with xylene and then stained with H&E. Three independent pathologists analyzed and reported on each section in a blinded manner.

### 5.8. Sample Preparation for LC-MS/MS Analysis

Lyophilized venom proteins (100 μg total) were reconstituted in 10 mM dithiothreitol (DTT) and incubated at 55 °C for 30 min. Subsequently, samples were cooled on ice to room temperature, followed by alkylation with 55 mM iodoacetamide (IAA) under dark conditions for 15 min at ambient temperature. Protein precipitation was achieved by adding six volumes of pre-chilled acetone and incubating at −20 °C for ≥4 h. The precipitates were pelleted via centrifugation (8000 rpm, 10 min, 4 °C). After evaporating the residual acetone for 2–3 min, pellets were resuspended in 100 μL of 50 mM ammonium bicarbonate (NH_4_HCO_3_) and digested with sequencing-grade trypsin (1 mg/mL, 1:100 *w*/*w*) overnight at 37 °C. Peptides were desalted using SOLA™ SPE 96-well plates (Thermo Fisher^®^, Waltham, MA, USA). Columns were activated with 200 μL methanol (triplicate) and equilibrated with 200 μL 0.1% aqueous formic acid (triplicate). Samples (500 μL) were loaded under vacuum at a 1 mL/min flow rate. After three washes with 200 μL of 0.1% formic acid, bound peptides were eluted thrice with 150 μL elution buffer (50% acetonitrile, 0.1% formic acid). Combined eluates (450 μL total) were vacuum-dried prior to the LC-MS/MS analysis [9,18,41].

### 5.9. LC-MS/MS Analysis

Prior to mass spectrometry, triplicate samples per species were spiked with iRT standards (1:20 ratio). Equal peptide amounts from digests were separated on a C18 analytical column (ThermoFisher^®^) using mobile phase A (0.1% formic acid/water) and B (0.1% formic acid/acetonitrile) at 400 nL/min with the gradient: 5–22% B (0–20 min), 22–37% B (20–24 min), 37–80% B (24–27 min), and 80% B (27–30 min). Peptides were analyzed on a timsTOF Pro mass spectrometer under these parameters: 1.4 kV capillary voltage, 180 °C dry gas temperature, 3.0 L/min dry gas flow, 100–1700 *m*/*z* MS scan range, 0.7–1.3 Vs/cm^2^ ion mobility range, and 20–59 eV collision energies. DIA raw data processing used Spectronaut Pulsar 18.4 against the UniProt_Elapidae database with 0.01 ppm precursor/0.01 Da protein mass thresholds, fixed carbamidomethylation (cysteine), variable modifications (methionine oxidation and *N*-terminal acetylation), ≤2 missed cleavages, and protein identification requiring ≥1 unique peptide. Protein identifications were validated using Morpheus scoring with homology-based alignment to exclude redundant peptides, and quantification was performed by extracting fragment ion chromatograms at the MS/MS level [9,18,41].

### 5.10. Statistical Analysis

All results were expressed as the mean ± standard error of the mean (SEM). All data were analyzed by one-way ANOVA followed by Tukey’s test (for multiple-group comparisons) or Student’s *t*-test (for two-group comparisons) using GraphPad Prism software (version 8, USA).

## Figures and Tables

**Table 1:** The LD_50_ of *N. atra* venom from different regions.

AH	GD	GX	HN	JX	YN	ZJ
0.51 mg/kg	0.31 mg/kg	0.27 mg/kg	0.48 mg/kg	0.45 mg/kg	0.23 mg/kg	0.63 mg/kg

**Table 2:** Identification of proteins in the venom of *N. atra* from different regions using liquid chromatography–tandem mass spectrometry (LC-MS/MS).

Category	Protein ID	Protein Name	Relative Quantification (Mean)						
			AH	GD	GX	HN	JX	YN	ZJ
**Metalloprotease**									
A0A024AXX7		p-III snake venom metalloprotease	/	0.063%	/	0.070%	/	0.054%	0.094%
A0A194ARL7		Metalloproteinase Type III	/	0.028%	0.028%	0.021%	/	0.019%	0.013%
D3TTC2		Zinc metalloproteinase-disintegrin-like atragin	2.801%	4.830%	4.221%	3.882%	3.364%	3.258%	3.258%
D5LMJ3		Zinc metalloproteinase-disintegrin-like atrase-A	0.405%	0.666%	0.575%	0.424%	0.696%	0.709%	0.345%
D6PXE8		Zinc metalloproteinase-disintegrin-like atrase-B	0.917%	2.003%	2.096%	1.359%	1.904%	1.578%	1.412%
E9JG34		Snake venom metalloproteinase	0.044%	0.053%	0.073%	0.040%	0.065%	0.109%	0.042%
P0DJ43		Zinc metalloproteinase-disintegrin-like mikarin	/	0.030%	/	/	/	0.045%	/
P82942		Hemorrhagic metalloproteinase-disintegrin-like kaouthiagin	2.159%	2.846%	2.689%	1.801%	1.788%	2.031%	1.862%
Q10749		Snake venom metalloproteinase-disintegrin-like mocarhagin	1.894%	1.617%	2.040%	1.229%	1.599%	1.175%	1.255%
Q2EI26		Snake venom metalloproteinase AaPA	/	0.005%	/	/	/	/	/
Q7LZS9		Snake venom metalloproteinase Ac1	/	0.019%	/	/	/	0.003%	/
Q9PVK7		Zinc metalloproteinase-disintegrin-like cobrin	/	0.009%	/	/	/	0.012%	0.011%
R4G2I1		Zinc metalloproteinase-Hop-23	/	0.003%	/	/	/	0.002%	/
**Phospholipase**									
A0A098LWY9		Phospholipase B-like	/	0.003%	/	/	/	0.006%	/
A0A898INR6		Phospholipase A2	0.017%	0.021%	0.035%	0.023%	0.024%	0.040%	0.033%
P00595		Basic phospholipase A2	/	0.044%	/	/	0.143%	0.070%	0.082%
P00596		Acidic phospholipase A2	3.027%	3.572%	3.507%	11.574%	4.284%	5.862%	3.931%
P00617		Basic phospholipase A2 beta-bungarotoxin A1 chain	/	/	/	/	/	0.000%	/
P00618		Basic phospholipase A2 beta-bungarotoxin A2 chain	/	/	/	/	/	0.000%	/
Q92084		Neutral phospholipase A2 muscarinic inhibitor	19.128%	20.183%	20.376%	25.987%	22.966%	41.201%	24.994%
**Oxidase**									
A0A0B8RQ82		Methanethiol oxidase	/	/	/	/	/	0.000%	/
A0A098LX00		Amine oxidase	/	0.006%	/	0.008%	/	0.009%	0.005%
A8QL58		L-amino-acid oxidase	0.645%	0.506%	0.577%	0.481%	0.529%	0.901%	0.512%
V8P7T9		Sulfhydryl oxidase	/	0.006%	/	/	/	0.006%	/
**Cysteine-rich secretory protein**									
A0A0F7Z2U7		Cysteine-rich secretory protein 1	0.181%	0.061%	/	0.122%	/	0.068%	0.093%
P0DL16		Cysteine-rich venom protein mossambin	0.602%	0.262%	0.266%	0.279%	0.321%	0.099%	0.138%
P84805		Cysteine-rich venom protein kaouthin-1	4.103%	3.623%	3.171%	2.594%	2.358%	3.744%	2.639%
P84808		Cysteine-rich venom protein kaouthin-2	0.138%	0.089%	0.134%	0.104%	0.105%	0.093%	0.088%
Q2XXP4		Cysteine-rich venom protein TRI1	0.135%	0.077%	0.092%	0.057%	0.069%	0.094%	0.060%
Q3SB03		Cysteine-rich venom protein pseudechetoxin-like	/	0.051%	0.030%	/	/	/	0.034%
E3P6P4		Cystatin	0.053%	0.050%	0.064%	0.070%	0.044%	0.065%	0.070%
**Muscarinic toxin-like protein**									
P0DQQ3		Muscarinic toxin-like protein Tx-NM3-2	/	0.108%	/	0.205%	0.226%	0.212%	0.101%
P82463		Muscarinic toxin-like protein 2	0.057%	0.108%	0.094%	0.111%	0.068%	0.132%	0.087%
P82464		Muscarinic toxin-like protein 3	/	1.586%	/	1.721%	/	2.068%	1.392%
**RNA processing and regulatory proteins**									
A0A0B8RTT7		40S ribosomal protein S10	/	/	/	/	/	0.008%	/
J3S9G0		60S ribosomal protein L18a	/	/	/	/	/	0.003%	/
V8NRR7		39S ribosomal protein L16, mitochondrial	/	/	/	/	/	0.000%	/
V8NTR1		Small ribosomal subunit protein uS5	/	/	/	/	/	0.001%	/
V8P8H9		Heterogeneous nuclear ribonucleoprotein A1 (Fragment)	/	0.003%	/	/	/	0.075%	0.010%
A0A0B8RX21		U6 snRNA-associated Sm-like protein LSm8	/	/	/	/	/	0.004%	/
A0A2D4G7G6		RRM domain-containing protein	/	0.004%	/	/	/	0.004%	0.023%
V8NZ75		Putative RNA-binding protein Luc7-like 2	/	/	/	/	/	0.002%	/
J3SC47		Elongation factor 2	/	/	/	/	/	0.005%	/
**Histone**									
V8NDF2		Histone H2A	0.008%	0.012%	/	/	/	0.002%	/
V8N8S2		Histone H4	/	0.007%	/	/	/	0.002%	/
**Metabolic enzymes**									
A0A0B8RST8		6-phosphogluconate dehydrogenase	/	/	/	/	/	0.002%	/
J3S119		Dihydrolipoyllysine-residue succinyltransferase component of 2-oxoglutarate dehydrogenase complex	/	0.001%	/	/	/	0.004%	/
V8NIF8		L-lactate dehydrogenase	/	0.001%	/	/	/	0.000%	/
**Resultant protein**									
A0A0B8RPH0		Vimentin	0.044%	0.023%	0.053%	0.041%	0.040%	0.020%	0.057%
A0A0F7Z8W5		Tubulin alpha chain	/	/	/	/	/	0.005%	/
J3SFJ0		Tubulin beta chain	/	/	/	/	/	0.026%	/
V8N9Y8		Tubulin alpha-8 chain	/	/	/	/	/	0.001%	/
A0A1W7RJI5		Alpha-actinin-1-like protein	/	/	/	/	/	0.000%	/
A0A0B8RYW7		Ezrin-like protein	/	0.012%	/	/	/	0.007%	/
A0A0B8RUG1		Katanin p60 ATPase-containing subunit A1	/	0.003%	/	/	/	0.001%	/
V8NBM9		Keratin, type II cytoskeletal 5	/	0.017%	/	/	/	0.002%	/
V8NYV8		Keratin, type II cytoskeletal 1	0.105%	0.082%	/	0.044%	/	0.019%	0.031%
V8P8L1		Keratin, type I cytoskeletal 19	0.234%	0.210%	0.204%	0.157%	0.195%	0.084%	0.131%
V8NL73		Extracellular matrix protein 1	/	0.021%	0.029%	/	0.015%	0.008%	0.025%
V8NRX1		IF rod domain-containing protein	0.187%	0.343%	0.136%	0.111%	0.157%	0.065%	0.082%
**Molecular chaperones and stress response proteins**									
V8N9M0		Heat shock protein HSP 90-alpha	/	/	/	/	/	0.026%	/
A0A0B8RUJ6		Glucose-regulated protein	/	0.033%	0.033%	/	/	0.003%	/
A0A1W7REY2		Protein disulfide-isomerase A6	/	0.040%	0.041%	/	/	0.003%	/
V8NBS9		Endoplasmic reticulum resident protein 44	/	0.005%	/	/	/	0.001%	/
J3SFD9		T-complex protein 1 subunit beta	/	/	/	/	/	0.001%	/
V8NWK2		T-complex protein 1 subunit epsilon	/	/	/	/	/	0.003%	/
**Immune and complement-related proteins**									
V8NCP4		Complement C3	0.379%	0.851%	0.473%	/	0.442%	0.538%	/
A0A1W7RH78		Gamma-interferon-inducible lysosomal thiol reductase	/	0.013%	/	/	/	0.011%	0.016%
D2YVI2		C-type lectin galactose-binding isoform	/	0.160%	/	0.190%	/	0.313%	0.132%
**3FTX**									
P01400		Weak toxin S4C11	0.436%	0.479%	0.607%	0.267%	/	0.288%	0.215%
P01401		Weak toxin CM-11	1.351%	1.516%	2.145%	0.817%	1.017%	1.646%	1.046%
P29181		Weak neurotoxin 7	0.731%	0.488%	0.548%	0.814%	0.707%	0.865%	0.524%
O42256		Weak neurotoxin 6	1.008%	1.073%	1.431%	0.556%	0.722%	1.052%	0.567%
P01424		Short neurotoxin 1	/	0.002%	/	/	/	0.002%	/
Q9YGI4		Probable weak neurotoxin NNAM2	2.112%	5.167%	2.550%	4.336%	3.549%	3.002%	3.128%
D5J9Q0		Non-conventional three finger toxin isoform 6	/	0.002%	/	/	/	0.002%	/
Q9W717		Neurotoxin-like protein NTL2	/	0.047%	/	/	/	0.056%	/
C0HJW9		Neurotoxin Nk-3FTx (Fragment)	0.037%	0.051%	0.050%	0.070%	0.073%	0.039%	0.069%
Q9DEQ3		Neurotoxin homolog NL1	/	/	/	/	/	0.000%	/
P34074		Long neurotoxin 1	0.086%	0.530%	0.594%	0.506%	0.691%	0.649%	0.467%
P60308		Cytotoxin SP15c	0.494%	1.784%	0.664%	1.180%	0.890%	0.506%	0.419%
Q91135		Cytotoxin I-like P-15	0.288%	0.464%	0.308%	0.150%	0.105%	0.090%	0.083%
P49122		Cytotoxin 7	/	0.249%	/	0.384%	0.166%	0.140%	0.343%
P80245		Cytotoxin 6	3.321%	0.063%	/	0.214%	0.160%	0.006%	0.041%
Q9W6W9		Cytotoxin 4N	0.476%	1.456%	0.543%	1.174%	0.879%	1.025%	0.686%
Q98962		Cytotoxin 3d	0.071%	0.025%	0.036%	0.018%	/	0.010%	0.010%
P01470		Cytotoxin 3	29.251%	20.677%	24.175%	15.939%	26.952%	6.192%	30.163%
P01440		Cytotoxin 2	4.710%	2.753%	3.893%	4.753%	5.856%	1.761%	3.813%
Q98956		Cytotoxin 1b	0.404%	0.088%	0.410%	0.201%	0.240%	0.054%	0.364%
P86541		Cytotoxin 10	0.196%	0.163%	/	0.168%	0.194%	0.120%	0.077%
P0CH80		Cytotoxin 1	1.567%	0.635%	1.487%	0.474%	0.483%	0.118%	0.432%
P59276		Cobrotoxin-c	/	0.034%	0.046%	0.008%	/	0.008%	0.011%
P59275		Cobrotoxin-b	0.132%	0.276%	0.330%	0.194%	0.108%	0.181%	0.194%
Q91126		Cardiotoxin 7a	0.532%	0.515%	0.408%	0.333%	0.439%	0.460%	0.239%
O57326		Alpha-neurotoxin NTX-3	0.025%	0.029%	0.029%	0.013%	0.017%	0.023%	0.012%
C0HM08		Alpha-elapitoxin-Nn2a	/	0.001%	/	/	/	0.000%	/
E2ITZ3		Alpha-elapitoxin-Na1a	1.318%	0.974%	1.032%	1.070%	0.781%	0.300%	0.631%
**Others**									
A0A2D0TC04		Venom phosphodiesterase	0.779%	0.467%	0.587%	0.726%	0.678%	0.712%	0.738%
V8NX10		WD repeat and FYVE domain-containing protein 1	/	0.013%	/	/	/	0.015%	0.017%
A0A098LYI7		Vespryn	/	/	/	/	/	0.012%	/
Q5YF89		Venom nerve growth factor	0.531%	0.497%	0.485%	0.515%	0.423%	0.767%	0.431%
A0A2D4Q7C6		Uncharacterized protein	/	/	1.022%	0.519%	/	0.753%	0.549%
A0A194AS98		Snake venom 5′-nucleotidase	0.752%	0.697%	0.897%	0.866%	0.707%	1.779%	0.954%
Q9DEF9		Snaclec anticoagulant protein subunit A	/	0.001%	/	/	/	/	/
B0FXL8		Siamenotoxin I	1.824%	1.750%	2.185%	0.853%	1.007%	0.693%	0.871%
V8NKT2		ShKT domain-containing protein	/	/	/	/	/	0.059%	/
A0A2D4HD83		SH3 domain-containing protein	/	0.003%	/	/	/	/	/
A0A2D4G403		SCP domain-containing protein	/	/	0.152%	/	/	/	/
C1IC50		Protease inhibitor 1	0.361%	0.195%	0.288%	0.266%	0.391%	0.063%	0.209%
A0A8C6VK71		Plasminogen activator	1.274%	0.929%	1.063%	1.133%	0.936%	1.145%	0.951%
A0A0B8RU52		Peptidyl-glycine alpha-amidating monooxygenase	/	0.003%	/	/	/	0.003%	/
A0A8C6Y6B8		Peptidase S1 domain-containing protein	/	0.006%	/	/	/	0.005%	/
I2C090		Ophiophagus venom factor	0.589%	1.238%	1.240%	0.892%	0.872%	1.341%	0.906%
V8P0W2		Neuroserpin	/	/	/	/	/	0.005%	0.004%
V8P1Y2		Neuroendocrine convertase 1	/	0.009%	/	/	/	0.006%	/
V8N4D8		Nerve growth factor-related domain-containing protein	2.396%	3.436%	2.331%	3.013%	2.043%	3.290%	2.833%
V8NQ76		Neprilysin	/	0.006%	/	/	/	0.004%	/
A0A2D4GN98		Multiple inositol polyphosphate phosphatase 1	0.046%	0.066%	0.072%	0.028%	0.042%	0.048%	0.018%
A0A898INP5		Kunitz peptide	/	/	/	/	0.035%	0.046%	0.036%
V8NNL9		Insulin-like growth factor-binding protein 3	0.011%	0.019%	0.017%	0.008%	/	0.010%	0.007%
A0A2D4LE84		Ig-like domain-containing protein	0.036%	0.061%	0.069%	0.079%	/	0.019%	0.164%
A0A898INC5		Hyaluronidase	/	0.003%	/	/	/	0.001%	/
A0A6J1VMA6		Hepatocyte growth factor activator	/	0.001%	/	/	/	0.002%	/
A0A2D4IPJ7		Granulins domain-containing protein	0.010%	0.010%	0.013%	0.009%	0.009%	0.011%	0.008%
V8P395		Glutathione peroxidase	0.676%	0.666%	0.775%	0.685%	0.559%	0.788%	0.786%
A0A2D4FFX4		GH18 domain-containing protein	/	0.005%	/	/	/	0.006%	/
U3FCT9		Endonuclease domain-containing 1 protein	/	0.256%	/	/	/	0.215%	0.072%
V8NG26		EH domain-containing protein 4	/	/	/	/	/	0.007%	/
A6MJH5		Dipeptidyl peptidase 4	/	0.004%	/	/	/	0.004%	0.006%
A0A346CI96		Cobra venom factor	/	/	0.143%	0.091%	/	0.084%	0.083%
A0A670ZPJ2		Coagulation factor VII	/	/	/	/	/	0.000%	/
J3SE58		Chromobox protein 3 like	/	/	/	/	/	0.015%	/
A0A0B8RRA8		Chitotriosidase	/	0.007%	/	/	/	0.006%	0.012%
U3FD65		Cathepsin B	/	0.004%	/	/	/	0.006%	/
P83346		Bucain	/	/	/	/	/	0.000%	/
A0A2D4GU19		BPTI/Kunitz inhibitor domain-containing protein	/	0.025%	/	0.038%	/	0.025%	0.032%
A0A6J1VTT9		BPTI/Kunitz domain-containing protein-like	0.032%	0.024%	0.023%	0.052%	0.038%	0.025%	0.038%
V8NEU2		B30.2/SPRY domain-containing protein	3.455%	2.526%	2.739%	1.919%	4.169%	1.970%	1.187%
A0A0B8RR92		ATP-dependent Clp protease ATP-binding subunit clpX-likeserine-threonine-like protein	0.005%	0.004%	0.006%	0.004%	/	0.004%	0.006%
U3FZS8		Aminopeptidase	/	0.017%	0.007%	/	/	0.017%	0.013%
V8ND09		Alpha-fetoprotein	/	0.013%	/	0.074%	/	0.027%	0.108%
A0A0B8RVP6		ADP/ATP translocase	/	/	/	/	/	0.001%	/
Q0ZZJ6		A.superbus venom factor 1	1.411%	3.127%	3.564%	1.888%	2.662%	2.504%	2.371%

## Data Availability

The original contributions presented in this study are included in the article. Further inquiries can be directed to the corresponding author(s).
